# Bone fragility in patients affected by congenital diseases non skeletal in origin

**DOI:** 10.1186/s13023-020-01611-5

**Published:** 2021-01-06

**Authors:** L. Masi, S. Ferrari, M. K. Javaid, S. Papapoulos, D. D. Pierroz, M. L. Brandi, IOF Skeletal Rare Diseases Working Group

**Affiliations:** 1 Metabolic Bone Diseases Unit, University Hospital of Florence, AOU-Careggi, Florence, Italy; 2 Division of Bone Diseases, Faculty of Medicine, Geneva University Hospital, Geneva, Switzerland; 3 Oxford NIHR Musculoskeletal Biomedical Research Unit, University of Oxford, Oxford, UK; 4 Center for Bone Quality, Leiden University Medical Center, Leiden, The Netherlands; 5 International Osteoporosis Foundation (IOF), Rue Juste-Olivier 9, 1260 Nyon, Switzerland; 6 Fondazione Italiana Ricerca sulle Malattie dell’Osso, Florence, Italy; 7 Department of Biomedical, Experimental and Clinical Sciences, University of Florence, Florence, Italy

**Keywords:** Bone metabolism, Genetic bone diseases, Metabolic bone diseases, Taxonomy, Non-skeletal rare congenital disorders

## Abstract

**Background:**

Bone tissue represents a large systemic compartment of the human body, with an active metabolism, that controls mineral deposition and removal, and where several factors may play a role. For these reasons, several non-skeletal diseases may influence bone metabolism. It is of a crucial importance to classify these disorders in order to facilitate diagnosis and clinical management. This article reports a taxonomic classification of non-skeletal rare congenital disorders, which have an impact on bone metabolism

**Methods:**

The International Osteoporosis Foundation (IOF) Skeletal Rare Diseases Working Group (SRD-WG), comprised of basic and clinical scientists, has decided to review the taxonomy of non-skeletal rare disorders that may alter bone physiology.

**Results:**

The taxonomy of non-skeletal rare congenital disorders which impact bone comprises a total of 6 groups of disorders that may influence the activity of bone cells or the characteristics of bone matrix.

**Conclusions:**

This paper provides the first comprehensive taxonomy of non-skeletal rare congenital disorders with impact on bone physiology.

## Background

In the past decade, spectacular progress has been made in the understanding of the molecular basis of congenital diseases and syndromes (https://www.eurordis.org/; www.orpha.net/). These mechanistic insights, in combination with the development of effective treatments, in a few cases engineered to target specific causes, have resulted in a significant increase in survival of the affected patients. A consequence of this longer life expectancy for patients, who previously died in infancy or at a very young age, is the development of signs and symptoms related to the genetic disorder and the related phenotype.

Bone tissue represents a large systemic compartment of the human body, with an active metabolism, that controls mineral deposition and removal. The dynamic process of bone turnover is the basis for a large number of local and systemic factors which control the function of bone cells [1]. This pleiotropic system is mirrored by an increasing prevalence of secondary osteoporosis, caused by a myriad of factors [2]. The recent classification of congenital metabolic bone disorders enabled identification of 116 Online Mendelian Inheritance in Man® (OMIM®) phenotypes with 86 affected genes, whose defects are based on recognized cellular and biochemical regulators of bone turnover [3].

In the present review, we report non-skeletal rare congenital diseases, which impact bone mass, bone quality and/or bone metabolism. The scope of this effort is to develop a taxonomic work in order to establish a platform for the recognition of characteristics and treatments of bone metabolic complications when these are the consequence of a non-skeletal rare congenital disease.

We are aware that to realize a complete classification is impossible. However, by this work we developed a first classification of systemic rare diseases not skeletal in nature, that influence bone metabolism. These facts open to the use of the drugs indicated for osteoporosis in areas not previously considered as pharmacological targets.

The present job will be revisited over time by the group of experts in order to update the paper and insert new disorders and potential drugs useful to treat the bone metabolic alterations.

## Methods

The present review was performed according to the following steps:

Step 1 involved a discussion by the scientists representing the IOF Skeletal Rare Diseases Working Group (SRD-WG) on the need to identify the non-skeletal rare congenital disorders which impact bone.

In step 2 the working group decided to identify relevant studies through a research strategy characterized by an evaluation on PUBMED. Systematic literature search on www.pubmed.gov using the term: Metabolic Rare Diseases, Liver Rare Diseases, Respiratory Rare Disease, Hematological Rare Diseases, Neurological Rare Diseases and Malformations. Inside to these big areas we selected the "congenital disorders".

In step 3, for each congenital disorders we added the term “osteoporosis” or “bone mass”, or “bone turnover” or “bone fragility” or “skeletal fractures”, or “bone biomarkers” or “biochemical”, “therapy” and we choose the most relevant studies.

Six groups of congenital disorders, which impact bone metabolism, were recognized. For each group a brief report regarding the pathogenesis of the disorders, the impact on bone metabolism was developed. The groups of diseases are described in Tables 1, 2, 3, 4, and 5.

**Table 1:** Metabolic rare disesases

Disease	OMIM phenotype number	OMIM gene/locus number	Gene	Chromosome location	Bone phenotype (specific signs and symptoms)	Bone biochemical markers
*A. Lysosomal storage diseases*						
*Aspartylglucosa-minuria*	208400	613228	*AGA*	4q34.3	Short stature, delayed skeletal maturation, kyphosis, scoliosis, flattening and anterior breaking of vertebral bodies, spondylolysis, spondylolisthesis, joint laxity, bursitis, pathologic fractures, mild dysostosis multiplex, and loss of weight (adult)	NR
*Gaucher disease, type I*	230800	606463	*GBA*	1q22	Gaucher cells in the bone marrow, growth retardation, osteolytic lesions, cortical thinning, osteonecrosis, osteosclerosis, bone crises, bone pain, osteopenia, osteoporosis, pathologic fractures, rarely acute osteomyelitis, vertebral compressions, avascular necrosis of femoral head, and erlenmeyer flask' deformity of the femurs	Markers of bone formation in treatment-naïve patients: N or low Markers of bone resorption: N or high.
*GM1-gangliosidosis, type III*	230650	611458	*GLB1*	3p22.3	Short stature, mild platyspondyly, bone dysplasia, mild anterior breaking of lumbar vertebrae, kyphosis, scoliosis, hypoplastic acetabulae, flat femoral heads, and flared iliac wings.	NR
*Scheie syndrome: mucopolysaccharidosis type IS; MPS1-S*	607016	607016	*IDUA*	4p16.3	Joint contractures, dysostosis multiplex, mild (in some patients), lumbar-sacral spondylolisthesis, genu valgum, carpal tunnel syndrome, claw-hand deformity, and pes cavus	NR
*Hurler- scheie syndrome: mucopolysaccharidosis IH/S*	607015	252800	*IDUA*	4p16.3	Short stature, dysostosis multiplex, joint stiffness, joint contractures, osteoporosis, scoliosis, kyphosis, progressive lumbar gibbus, and flat femurs with short metaphyses	High serum BSAP and OCN High urine DPD Low serum 25 (OH) D_3_
*Mucopolysaccharidosi* *type II; MPS2* *(hunter syndrome)*	309900	300823	*IDS*	Xq28	Mild dwarfism, adult height 120-150 cm, scaphocephaly, macrocephaly, stiff joints, joint pain, restrictive joint range of motion, hip dysplasia, arthropathy, dysostosis multiplex, osteoporosis, kyphosis, flexion contractures, claw hand, and pes cavus.	High serum BSAP and OCN High urine DPD Low serum 25 (OH) D_3_
*Mucopolysaccharidosis type IVA; MPS4A* *(morquio syndrome A)*	253000	612222	*GALNS*	16q24.3	Short stature (adult height 82 to 115 cm), short-trunked dwarfism, pectus carinatum, osteoporosis, platyspondyly, odontoid hypoplasia, cervical subluxation, kyphosis, ovoid vertebral bodies, hyperlordosis, scoliosis, coxa valga, constricted iliac wings, laxity of wrist joints, genu valgum, ulnar deviation of the wrist, epiphyseal deformities of tubular bones, and widened metaphyses.	NR
*Mucopolysaccharidosis type IVB; MPS4B (morquio syndrome B)*	253010	611458	*GLB1*	3p22.3	Short stature (adult height 82-115 cm), short-trunked dwarfism, osteoporosis, platyspondyly, odontoid hypoplasia, cervical subluxation, kyphosis, hyperlordosis, scoliosis, ovoid vertebral bodies, coxa valga, constricted iliac wings, joint laxity, severe genu valgum, ulnar deviation of the wrist, epiphyseal deformities of tubular bones, and widened metaphyses.	NR
*Multiple sulfatase deficiency; MSD*	272200	607939	*SUMF1*	3p26.1	Short stature, dysostosis multiplex, hypoplastic vertebral bodies, broad thumbs, and broad index fingers.	NR
*Mucolipidosis II alpha/beta (I-cell disease; ICD)*	252500	607840	*GNPTAB*	12q23.2	Birth length less than normal, deceleration of linear growth during first year**, **marked growth retardation**, **moderate joint limitation, scapular hypoplasia, dysostosis multiplex, osteopenia in early infancy, pathologic fractures**, **thickened cranium, craniosynostosis, dorsolumbar kyphosis, atlantoaxial dislocation, ovoid vertebral bodies, narrowness of interpediculate distances in lower thoracic regions, hypoplastic odontoid process, beaking of vertebral bodies T12-L3, lumbar gibbus**, **flared iliac wings, horizontal acetabular roofs, supra-acetabular constriction, hip dislocation, irregular contours of pubis and ischium**, **cortical bone erosion (especially proximal femora), long bone shortening, widened metaphyses, varus deformity of humeral neck, tilted distal ends of radius and ulna**, **broadening of wrist, brachyphalangia, hypoplasia of carpal bones, conical bullet-shaped distal ends of phalanges, claw-hand deformities, and talipes equinovarus	NR
*Mucolipidosis III alpha/beta (pseudo-hurler polydystrophy)*	252600	607840	*GNPTAB*	12q23.2	Short stature, short and thick clavicles, wide and slightly short ribs**, **dysostosis multiplex, scoliosis, absence of dens, vertebral beaking**, **flaring of iliac wings, shallow acetabular fossae**, **shoulder stiffness, broad metaphyses, short long bones**, **hand stiffness, claw-hand deformities, carpal tunnel syndrome, soft tissue swelling of interphalangeal joints, and small and irregular carpal bones**.**	NR
*Mucolipidosis II alpha/beta (I-cell disease; ICD)*	252500	607840	*GNPTAB*	12q23.2	Birth length less than normal, deceleration of linear growth during first year**, **marked growth retardation**, **moderate joint limitation, scapular hypoplasia, dysostosis multiplex, osteopenia in early infancy, pathologic fractures**, **thickened cranium, craniosynostosis, dorsolumbar kyphosis, atlantoaxial dislocation, ovoid vertebral bodies, narrowness of interpediculate distances in lower thoracic regions, hypoplastic odontoid process, beaking of vertebral bodies T12-L3, lumbar gibbus**, **flared iliac wings, horizontal acetabular roofs, supra-acetabular constriction, hip dislocation, irregular contours of pubis and ischium**, **cortical bone erosion (especially proximal femora), long bone shortening, widened metaphyses, varus deformity of humeral neck, tilted distal ends of radius and ulna**, **broadening of wrist, brachyphalangia, hypoplasia of carpal bones, conical bullet-shaped distal ends of phalanges, claw-hand deformities, and talipes equinovarus	NR
*Mucolipidosis III alpha/beta (pseudo-hurler polydystrophy)*	252600	607840	*GNPTAB*	12q23.2	Short stature, short and thick clavicles, wide and slightly short ribs**, **dysostosis multiplex, scoliosis, absence of dens, vertebral beaking**, **flaring of iliac wings, shallow acetabular fossae**, **shoulder stiffness, broad metaphyses, short long bones**, **hand stiffness, claw-hand deformities, carpal tunnel syndrome, soft tissue swelling of interphalangeal joints, and small and irregular carpal bones**.**	NR
*Fabry disease*	301500	301644	#301500	Xq22.1	Hands with limited extension of terminal joints, osteoporosis, non–traumatic fractures	High serum PTH and OCN
*B. Disorders of sulfur metabolism*						
*Homocystinuria* *(due to cystathionine beta-synthase deficiency)*	236200	613381	*CBS*	21q22.3	Normal to tall stature, generalized osteoporosis, biconcave 'codfish' vertebrae, kyphoscoliosis, dolichostenomelia, arachnodactyly, and limited joint mobility	NR
*C. Disorders of tyrosine pathway*						
*Alkaptonuria*	203500	607474	*HGD*	3q13.33	Height loss secondary to spinal changes, joint ochronosis and subsequent osteoarthritis (ochronotic pigmentation of fibrous tissues including cartilage, tendons, ligaments, intervetebral disks, ochronotic arthritis and arthropathy), chronic joint pain, back pain, kyphosis, degeneration of intervertebral disks, fusion of vertebral bodies, osteopenia, osteoporosis, fractures, spondylosis, peripheral arthropathy, decreased lumbar flexion and tendon and ligament ruptures	High serum NTx and BSAP
*Phenylketonuria*	261600	612349	*PAH*	12q23.2	Low bone mineral density	In children: low serum BSAP, OCN, and P1NP In adults: high urine PYR, serum CTX, and NTx and low serum OPG

**Table 2:** Liver rare diseases

Disease	OMIM phenotype number	OMIM gene/locus number	Gene	Chromosome location	Bone phenotype (specific signs and symptoms)	Bone biochemical markers
*Disorders of copper pathway*						
*Menkes disease*	309400	300011	*ATP7A*	Xq21.1	Intrauterine growth retardation, failure to thrive, short stature, Wormian bones, osteoporosis, joint laxity, and metaphyseal widening with spurs A milder form is occasionally seen in males with mild intellectual disability, muscle weakness, tremor, ataxia, connective tissue signs, pili torti, and later-onset seizures	NR
*Wilson disease*	277900	606882	*ATP7B*	13q14.3	Osteoporosis, osteomalacia, pathological fractures, chondrocalcinosis, osteoarthritis, and joint hypermobility	High urine phosphate and calcium Low serum calcium Low PTH

**Table 3:** Respiratory rare disease

Disease	OMIM phenotype number	OMIM gene/locus number	Gene	Chromosome location	Bone phenotype (specific signs and symptoms)	Bone Biochemical Markers
*Cystic fibrosis*	219700	602421	*CFTR*	7q31.2	Failure to thrive, defective height-weight growth, osteoporosis, non-traumatic fractures	High serum CTX Low serum 25(OH)D_3_

**Table 4:** Hematological rare diseases

Disease	OMIM phenotype number	OMIM gene/locus number	Gene	Chromosome location	Bone phenotype (specific signs and symptoms)	Bone biochemical markers
*Mastocytosis*	154800	164920	*KIT*	4q12	Osteopenia, osteoporosis with/without fractures osteolytic or osteosclerotic bone lesions due to mast cell infiltration, primarily affecting the axial skeleton and ends of the long bone	High serum BSAP and urine DPD
*Beta-thalassemia major*	613985	141900	*HBB*	11p15.4	Osteopenia and osteoporosis with/or without fractures	Low PTH and 25(OH)D_3_ High serum IL-1α, TNF-α and IL-6 Low serum OCN
*Hemophilia* Type A	306700	300841	*F*	Xq28	Osteoporosis	High serum P1NP
*Hemophilia* *Type B*	306900	300746	*F9*	Xq27.1	Osteoporosis	High serum P1NP
*Sickle cell disease*	603903	141900	*HBB*	11p15.4	Avascular joint necrosis; Joint and leg pain; osteopenia/osteoporosis	Low serum calcium and high serum phosphate, High serum BSAP
*Ghosal* *hemato-diaphyseal dysplasia *	231095	274180	*TBXAS1*	7q34	Increased bone density; diaphyseal dysplasia; thick long bones of the extremities; wide diaphyseal medullary cavities; cortical hyperostosis Variable phenotype; most patients present in infancy with anemia	NR
*Severe congenital neutropenia 1*	202700	130130	*ELANE*	19p13.3	Osteopenia/osteoporosis	NR
*Severe congenital neutropenia 2*	613107	600871	*GFI1*	1q22.1	Osteopenia/osteoporosis	NR
*Severe congenital neutropenia 3*	610738	605998	*HAX1*	1q21.3	Osteopenia/osteoporosis	NR
*Severe congenital neutropenia 4*	612541	611045	*G6PC3*	17q21.31	Osteopenia/osteoporosis; poor growth; pectus carinatum; proximal localization of thumb; broad thumbs	NR
*Severe congenital neutropenia 5*	615285	610035	*VPS45A*	1q21.2	Osteopenia / osteoporosis	NR
*Severe congenital neutropenia 6*	616022	616012	*JAGN1*	3p25.3	Short stature; osteopenia / osteoporosis	NR
*Severe congenital neutropenia 7*	617014	138971	*CSF3R*	1p34.3	Osteopenia/osteoporosis	NR
*Severe congenital neutropenia x-linked*	300299	300392	*WAS*	Xp11.23	Osteopenia/osteoporosis	NR
*Histiocytosis*						
* Langerhans cell histiocytosis (X-Histiocytosis*					Lytic bone lesions at skull, but any bone may be involved, painless or painful, and possible associated soft-tissue mass.	NR
* Non-Langerhans cell histiocytosis (Erdheum-Chester Disease)*					Osteosclerosis of the long bones, bone pain, (mainly affecting the distal lower limbs).	NR

**Table 5:** Neurological rare diseases

Disease	OMIM phenotype number	OMIM gene/locus number	Gene	Chromosome location	Bone phenotype (specific signs and symptoms)	Bone biochemical markers
*Rett Syndrome*	312750	300005	*MECP2*	Xq28	Short stature, deceleration of head growth, scoliosis, and growth retardation, kyphosis, small feet, peripheral vasomotor disturbance, muscle wasting, usually low bone mineral density, high risk of fractures The evolution and severity of the disease are heterogeneous and several atypical variants were observed	Low bone turnover in the modeling period of childhood and youth, normal bone turnover in adults with the exception of higher serum BSAP

Acronyms for the disorders were described with phenotype and gene/locus numbers presented according to the nomenclature of Online Mendelian Inheritance in Men® (OMIM®) database, as accessed on July 2014 (https://www.ncbi.nlm.nih.gov/omim). An OMIM entry preceded by a number sign (#) indicates the phenotype and specific OMIM entries for the genes/loci whose mutations have been shown as responsible for that phenotype (https://web.udl.es/dept/cmb/biomatica/OMIM.PDF). The names of genes/loci are those approved by HGNC (HUGO Gene Nomenclature Committee, https://www.genenames.org).

The scope of the present paper is to classify non-skeletal rare congenital disorders with an impact on bone physiology on the basis of phenotypes. Information of bone metabolism in these disorder is also included. The manuscript was conceived and written by the members of the IOF SRD-WG.

The diseases have been homogenously described following this scheme:

- Description of the systemic disease
- Genetic defect
- Pathophysiology of bone phenotype
- Therapy

A brief description of the bone phenotype and altered biomarkers, when available, has been reported for each disease and for cases for which this information missing the term “NR” (not reported) is included.

The therapy for the systemic disease has been described if available and studies on drugs used to treat the bone damage were present in the literature.

The alteration of bone metabolism requires a particular attention considering that, due to better targeted therapies, patients survive into adulthood and in many cases the loss of bone mass may be an important problem with an impact on the quality of life.

An adequate patient education in term of intake of calcium, vitamin D and physical activity should be recommended. Currently, the best advice is to treat osteopenia and/or osteoporosis according to general guidelines using anti-resorbitives or anabolic drugs.

**Fig. 1:**
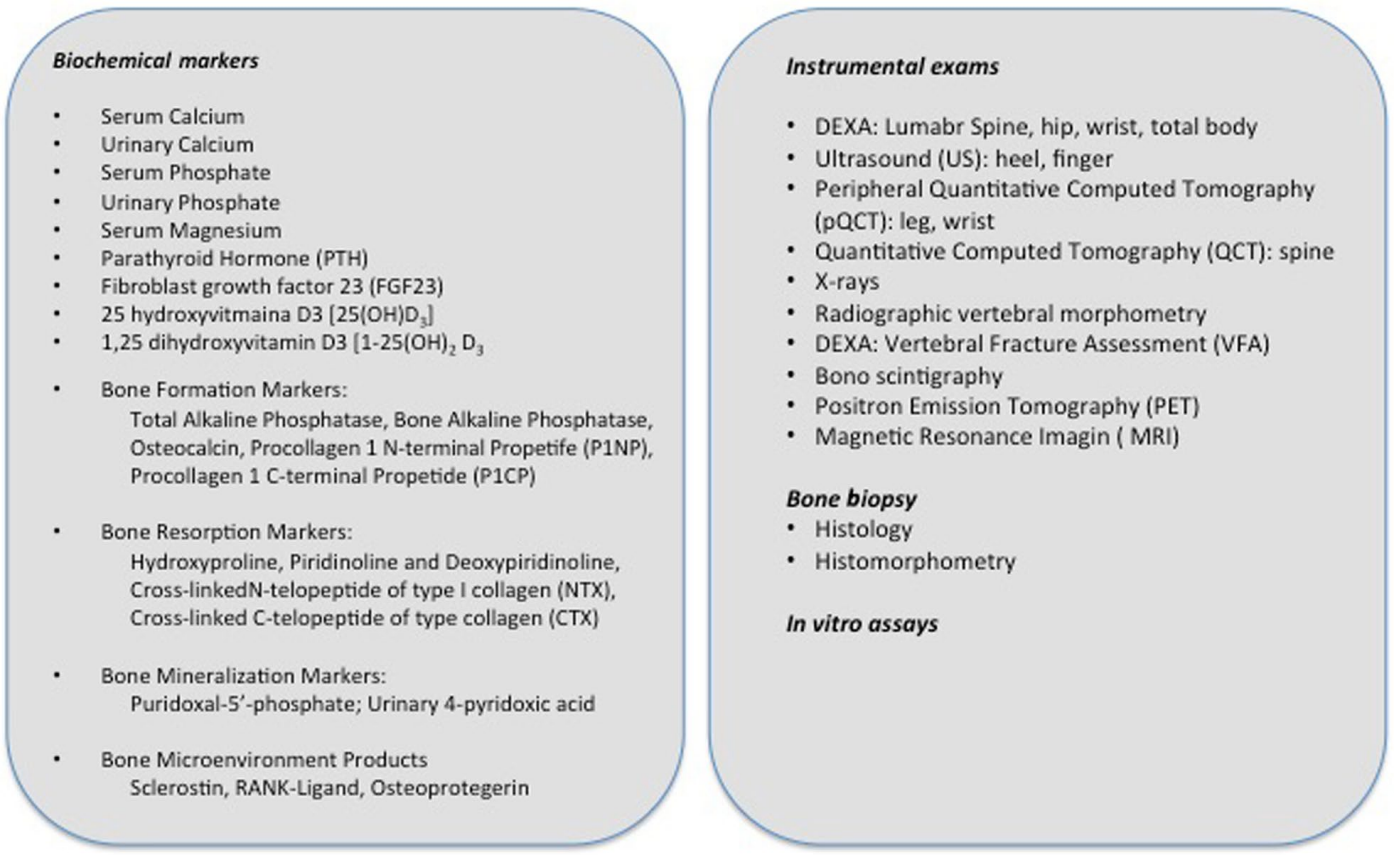
Biochemical/instrumental exams and in vitro tests for characterizing metabolic bone involvement [3]

In Fig. 1, the available non disease-specific diagnostic assays, which could be of use to further refine the diagnosis, have been listed, as previously described [3].

## Metabolic rare diseases

### Lysosomal storage diseases

#### Description of the systemic disease

The identification of the lysosome as a cellular organelle responsible for intracellular digestion and recycling of macromolecules [4–7] led to the understanding of the physiological basis of the Lysosomal Storage Diseases (LSDs), as a heterogeneous group of over 50 rare inherited disorders characterized by the accumulation of undigested or partially digested macromolecules, which ultimately results in cellular dysfunction and clinical abnormalities [8]. The constellation of dysmorphic features also includes bony abnormalities (dysostosis multiplex) [8]. Symptoms are typically gradually progressive rather than episodic, as occurs with other neurometabolic disorders.

Classically, LSDs encompass enzyme deficiencies of the lysosomal hydrolases. A classical LSD is Type 1 Gaucher disease (GD) a β-glucocerebrosidase deficiency, which is a relatively common LSD, particularly within the Ashkenazi Jewish community. In 1882, it was the first of these disorders to be described [9], followed by Fabry disease in 1898 [10].

More recently, the concept of lysosomal storage disease has been expanded to include deficiencies or defects in proteins necessary for the normal post-translational modification of lysosomal enzymes (which themselves are often glycoproteins), activator proteins, or proteins important for proper intracellular trafficking between the lysosome and other intracellular compartments [11]. Age of onset and clinical manifestations may vary widely among patients with a given lysosomal storage disease, and significant phenotypic heterogeneity between family members carrying identical mutations has been reported [12]. LSDs are generally classified by the accumulated substrate and include the sphingolipidoses, oligosaccharidoses, mucolipidoses, mucopolysaccharidoses (MPSs), lipoprotein storage disorders, lysosomal transport defects, neuronal ceroid lipofuscinoses, and others.

#### Genetic defects

In each case, lysosomal storage diseases are caused by an inborn error of metabolism that results in the absence or deficiency of an enzyme, leading to the inappropriate storage of material in various cells of the body. While most lysosomal storage disorders are inherited in an autosomal recessive pattern, there are exceptions. Fabry disease and Hunter syndrome follow an X-linked recessive inheritance pattern. The genes associated with many lysosomal storage disorders have been identified. The genes involved in the pathogenesis of the disorders and the chromosome location are indicated in Table 1A.

#### Pathophysiology of bone phenotype

Lysosomes have been isolated from cartilage, bone and synovial tissues. In pathological conditions, increased numbers of lysosomes have been observed by super resolution microscopy and electron microscopy in animal and human tissues [13], in particular rheumatoid synovium [13] and experimentally produced arthritis [14].

The in vitro studies showed that the initial degradation of extracellular matrix is due, at least in part, to the extracellular function of lysosomal enzymes released from viable cartilage and bone cells [13]. Further, it was shown that this initial degradation allowed diffusion of products out of the matrix and stimulated their endocytosis by chondrocytes, osteocytes and fibroblasts, and the digestion is completed within the digestive vacuoles of these cells. Extracellular digestion that takes place during the bone erosion process is due to the activity of osteoclastic cells, characterized by the presence of a large number of lysosomes [13], where enzymes, when released on bone surfaces, are able to degrade the extracellular matrix. It is, therefore, not surprising that the majority of LSDs have bone involvement. It occurs frequently in GD patients, in which it is one of most debilitating complication, reducing the quality of life of patients [15, 16]. However, in GD patients, the pathogenic basis of bone complications is not fully understood [16]. The need for a routine analysis of bone turnover, bone mass and bone quality features is strongly recommended in these patients, as this will facilitate pharmacological intervention preferentially with antiresorptives and/or targeted metabolic drugs.

#### Therapy

The enzyme replacement therapy (ERT) often fails to adequately correct the manifestations of the disease in the CNS and other organs such as bone, cartilage, cornea, and heart. Targeted enzyme delivery systems (EDSs) can efficiently cross biological barriers such as the blood–brain barrier (BBB) and provide maximal therapeutic results with minimal side effects, and hence, offer great clinical benefits over the currently used conventional ERT. The classical ERT has been beneficial in MPS patients, improving their survival rate. However, some difficulties have impaired the therapeutic values of ERT, including the inability of enzymes in crossing the BBB, their low stability, and the requirement for an early treatment to prevent the occurrence of irreversible clinical manifestations. Thus, it is essential to use less aggressive and more effective treatment modalities in newborn patients to avoid more-aggressive interventions later in life. Multifunctional nanomedicines have been introduced as the novel strategies to circumvent these limitations and increase the effectiveness of the ERT. Nano-scaled liposomes and polymers conjugated with specific cell surface receptors provide a new approach in targeting the desired cells. This strategy is still in its infancy period, however it could provide a cost-effective approach with high rate of success [17].

In Table 1A, LSD disorders are described, along with accompaining systemic and bone signs and symptoms.

### Disorders of sulfur metabolism

#### Homocystinuria

##### Description of the systemic disease.

Several defects can exist in the conversion of the sulfur-containing amino acid methionine to cysteine and the ultimate oxidation of cysteine to inorganic sulfate [18]. The most relevant disorder is rapresented by homocystinuria. Cystathionine-β-synthase (CBS) is a crucial regulator of plasma concentrations of homocysteine (HCY) [18]. It catalyzes the pyridoxal 5′-phosphate (PLP)-dependent’-replacement reaction in which the thiolate of L-homocysteine replaces the hydroxyl group of L-serine [19]*.* CBS is an especially interesting PLP enzyme because it has a complex domain structure and regulatory mechanism. The allosteric activator, S-adenosyl-L-methionine (AdoMet), increases CBS activity about threefold, and likely binds to the C-terminal regulatory domain [19]*.*

The clinical features of untreated homocystinuria due to CBS deficiency usually manifest in the first or second decade of life and include myopia, ectopia lentis, mental retardation, skeletal anomalies resembling Marfan syndrome (MFS), and thromboembolic events. Light skin and hair can also be present. It is extremely heterogeneous, ranging from patients presenting with all of the complications to individuals with no overt clinical involvement. Biochemical features include increased urinary homocystine and methionine. There are 2 main phenotypes of the classic disorder: a milder pyridoxine (vitamin B6)-responsive form, and a more severe pyridoxine-nonresponsive form. Pyridoxine is a cofactor for the CBS enzyme, and can aid in the conversion of homocysteine to cysteine [20–22].

##### Genetic defects

Classic homocystinuria is an autosomal recessive metabolic disorder of sulfur metabolism. The human hereditary disease is characterized by very high plasma levels of the toxic amino acid L-homocysteine. The gene coding CBS is localized on chromosome 21q22.3. A large number of mutations in different regions of the human CBS have been found in patients with homocystinuria [19, 23, 24]. Mutations in the CBS gene can alter the mRNA stability, the enzyme activity, the binding of PLP and heme and the allosteric regulation. Rarely, homocystinuria can be caused by mutations in other genes. The enzymes encoded by the *MTHFR, MTR, MTRR*, and *MMADHC* genes play a role in converting homocysteine to methionine [23–25].

##### Pathophysiology of bone phenotype

Severe hyperhomocysteinemia due to CBS deficiency confers diverse clinical manifestations, including typical skeletal abnormalities. This aspect of hyperhomocysteinemia has been investigated in the skeleton of *cbs*-deficient mice, a murine model of severe hyperhomocysteinemia, characterized by impaired cartilage differentiation, albeit to differing degrees [25, 26]. Interestingly, basal levels of *cbs* gene mRNA and protein in MC3T3-E1 murine pre-osteoblasts appear to increase after incubation with 1,25-dihydroxyvitamin D_3_ [1,25(OH)_2_D_3_] providing evidence of a transcriptional regulation of the vitamin D receptor (VDR) [26], with a possible supporting role for calcitriol on the action of folic acid, vitamin B6, and B12 in lowering high HCY levels [27].

Some of the skeletal abnormalities, are due to the fact that cysteine deficiency, which occurs in hyperhomocysteinemia due to CBS deficiency, can induce a fibrillin-1 defect [28].

Because collagen cross-links are important for the stability and strength of the bone matrix, CBS deficiency patients are prone to fragile bone [29]. In addition, homocysteine levels increase the risk of fractures, independent of bone mineral density (BMD) and other potential risk factors for fracture [29, 30], likely for a disruption of a normal microfibrillar configuration [22].

##### Therapy

For early diagnosed patients, treatment can realistically aim to prevent all the complications of CBS deficiency, as normal growth and nutrition, allowing the patient normal opportunities for employment and family life. For late-diagnosed patients, the aim is to prevent further complications, especially thromboembolic disease. Good compliance with low dietary methionine restriction treatment prevented ectopia lentis, osteoporosis and thromboembolic events and it also led to normal cognitive functions. Enzyme replacement therapy based on polyethylene glycol (PEG)ylated 20NHS CBS conjugate represents the most suitable candidate for manufacturing and clinical development [31].

Table 1B describes only homocystinuria phenotype disease as the main disorder of sulfur metabolism, where bone involvement has been described.

### Disorders of tyrosine metabolism

#### Description of the systemic disease

Tyrosine is one of the 20 standard amino acids that are used by cells to synthesize proteins. Phenylalanine hydroxylase (PAH) converts phenylalanine to tyrosine. Tyrosine can also be found in dairy products, meats, fish, eggs, nuts, beans, oats, and wheat. Tyrosine is a precursor of several neurotransmitters (e.g. dopamine, norepinephrine, epinephrine), hormones (e.g. thyroxine), and melanin. Deficiency of enzymes involved in its metabolism leads to a variety of syndromes. Alkaptonuria and phenylketonuria are the two main diseases due to a tyrosine pathway alteration with an impact on bone metabolism.

#### Alkaptonuria

##### Genetic defects

Alkaptonuria is an inherited disorder of aromatic amino acid metabolism and results from absence of homogentisate 1,2 dioxygenase (HGD), the enzyme predominantly produced by hepatocytes in the liver and in the kidney, responsible for the breakdown of homogentisic acid (HGA). Deficient HGD activity within the liver causes HGA levels to rise systemically. Large (gram) quantities of HGA are removed by urinary excretion on a daily basis [32, 33].

It is an autosomal recessive genetic disease, meaning that patients have inherited two defective copies of a gene; one from each parent. By 1995, the genetic defect was discovered, cloned, and mapped to chromosome 3 between regions 3q21-q23  [32–34].

##### Pathophysiology of the bone phenotype

The development of transgenic mice and in vitro models have enabled a better understanding of the pathophysiology involved in the progression of ochronosis and the related osteoarthropathy. Research has identified that HGA is present in healthy cartilage but becomes susceptible to degeneration only following focal changes [35]. Indeed, extracellular matrix (ECM) is normally resistant to ochronosis, but may become susceptible to pigmentation in response to tissue biochemical or mechanical damage, including microtrauma [35]. Pigmentation appears to protect collagen fibers from the action of proteolytic enzymes, contributing to increased tissue stiffness. This is a probable cause of higher susceptibility to matrix damage through normal loading [35]. Pigmentation is shown to begin in the pericellular and territorial matrices of individual chondrocytes and, thus, matrix turnover events in these regions could be an important factor for initiation of pigmentation into the hyaline cartilage [35, 36]. This process of enhanced osteoclastic activity resorbing the unloading subchondral bone and calcified cartilage is similar to the manifestation reported in osteoarthritis [37–40]. Although aggressive resorption appears to be focal, it is noteworthy that enhanced urinary excretion of crosslinked N-telopeptides of type I collagen has been reported in alkaptonuria patients [34]. In addition, Aliberti et al. [41] showed an increase of bone resorption along with an almost normal bone formation in patients with alkaptonuria, suggesting an enlarged bone remodeling space. In term of bone mass, they found conflicting results about spinal and femoral BMD, as lumbar spine BMD was normal or markedly increased, whilst femoral neck BMD was almost always reduced [41]. It is likely that BMD of lumbar spine might not be reliably assessed in these particular patients. It may be hypothesized that the homogentisic acid polymer deposit in the bone matrix and osteocytes may play a pathophysiological role in accelerating bone loss [41]. It is plausible that in a bone tissue diffusely osteoporotic, the ochronotic pigment is deposited in bone matrix and osteocytes with degenerated or dead cells [41]*.*

##### Therapy

In a phase 3 study patients with AKU were given 2 mg of nitisinone each day for three years. Newly published results show that the drug stops the disease, by decreasing HGA. Further, nitisinone therapy not only arrested but also partially reversed ochronosis. Results also show that it significantly reduces the damage caused by ochronosis, especially in the joints. Patients who took nitisinone showed major health benefits [42].

#### Phenylketonuria

##### Genetic defects

Phenylketonuria (PKU) is a genetic disorder caused by mutations in the gene coding for PAH. As a consequence, the essential amino acid phenylalanine (Phe) cannot be converted to tyrosine and accumulates in the blood.

The human *PAH* gene, which is located on chromosome 12q, consists of 13 exons spanning 90 kb. To date, more than 520 different mutations in the *PAH* gene have been characterized in PKU patients and recorded in the *PAH* Mutation Analysis Consortium Database (https://www.pahdb.mcgill.ca). Although most of these mutations are detectable by sequence analysis, with a detection rate of 95%, large intragenic deletions or duplications cannot be identified using this method. In these cases, multiplex ligation-dependent probe amplification (MLPA) can be used as a sensitive and efficient method [43, 44]*.*

##### Pathophysiology of the bone phenotype.

Bone complications are seen in early and continuously treated patients [44]. Indeed, most studies on patients with PKU indicate that bone is often affected. However, there are significant gaps in the pathogenetic basis of this complication neither consensus exists on the degree and implications of bone abnormalities and the risk factors for low BMD [4, 5, 14]. To investigate these gaps, a meta-analysis on BMD, corrected for bias, age and gender, has been performed by Rucker RB et al. [45]. The Authors found that BMD in early diagnosed and treated patients with PKU is below the healthy population average but within the normal range [45]. Moreover, data on a corresponding higher risk of fracture in these patients are missing [45].

Other indicators of bone status in early treated patients with PKU are inconclusive due to the small number of studies and the heterogeneity in the groups examined and in the measurement methods. A recent meta-analysis found that low BMD does not seem to be an exaggerated concern in patients with PKU, and that research is needed on the effects of the PKU diet on bone, on the reliability of bone turnover markers in bone assessment, and on a concrete estimate of fracture risk in patients with PKU [45].

##### Therapy

Dietary treatment is the basis of PKU management. It consists of 3 components: natural protein restriction, Phe-free-L-amino acid supplements, and low protein food intake. Effectiveness of PKU treatment is demonstrated by any of the following objective measures: reduction in Phe blood concentrations, increase in natural protein tolerance, improvement in neuropsychological testing, in the nutritional status, and in the quality of life [46]. A possible enzyme replacement therapy using PEG Phenylalanine-Ammonia Lyase (PAL) or Pegvaliase is under investigation [46]. PEG-PAL trials have proven short-term reduction in the Phe blood concentrations in adult PKU patients, but further studies are required to observe long-term effectiveness and safety. Results of a phase III extension study (NCT01819727) are awaited. Gene therapy and therapeutic liver repopulation have been investigated in murine models only, and larger animal PKU models and human studies need to be developed [46].

Table 1C describes the diseases due to the alteration of tyrosine pathway in which the skeletal involvement has been described.

## Liver rare diseases

### Disorders of copper metabolism

Under the liver rare diseases we selected the diseases with a main impact on bone, being the ones characterized by disorders of Copper metabolism.

#### Description of the systemic disease

**Fig. 2:**
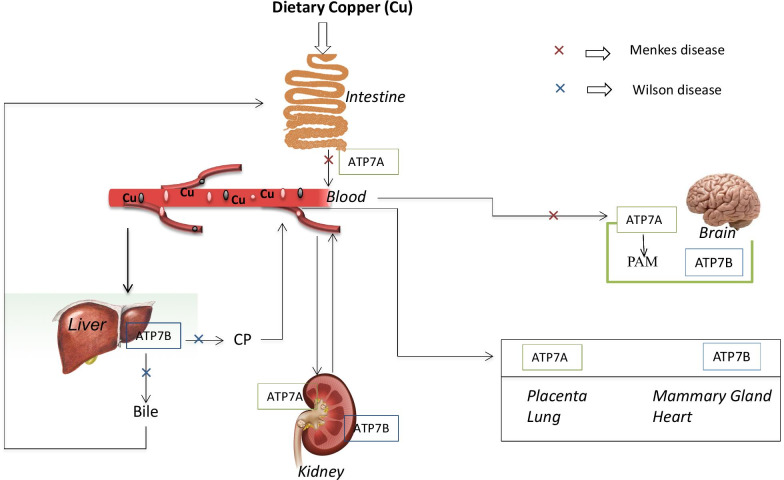
Distribution of Cu in the body. Most endogenous Cu is lost via bile, being excreted into the gastrointestinal tract. Biliar Cu combines with small amounts of Cu from pancreatic and intestinal fluids and intestinal cells and is eliminated from the body. Whole-body Cu retention increases with increased dietary intake. Little Cu is lost in the urines. The highest Cu concentrations are in the liver, brain, kidney, and heart. ATP7A: Cu is exported from the enterocytes into the blood by Cu-ATPase (ATP7A). ATP7B: export of Cu from the liver is mediated by a copper-transporting Cu-ATPase (ATP7B). CP: portal circulation

Copper (Cu) has an essential role in the normal maturation of collagen, particularly in the important steps of the formation of lysine-derived cross-links [47]. Since 1950, several reviews on Cu metabolism have contributed to the understanding of the human whole-body Cu metabolism [48–50]. The reviews generally reached similar conclusions on many aspects of Cu metabolism [48–50]. Dietary Cu is absorbed into the body through the intestinal mucosa and transported via the portal blood to the liver, where it is prevalently incorporated into ceruloplasmin, released into the blood, and delivered to tissues. The Cu uptake into the liver does not appear to be highly regulated. In contrast, the export of Cu from the liver is a regulated Cu-dependent process, which is mediated by a Cu-transporting Cu-ATPase ATP7B. Cu is exported from the enterocytes into the blood by Cu-ATPase ATP7A in a process that involves trafficking of the transporter towards the baso-lateral membrane [51]. Details of these mechanisms and cellular metabolism are covered in Fig. 2.

In humans, Cu is characterized by very low storage in the body (< 100 mg) compared with other trace elements such as zinc and iron [48]. The highest Cu concentration is in the liver, followed by the brain, kidney and heart [48]. Several conditions and disorders influence whole-body Cu metabolism, including metabolic defects, such as Menkes syndrome and Wilson disease, pregnancy, inflammation, and numerous other diseases [48]. Blood concentrations increase dramatically in many of these conditions and several tissues, including bone, are damaged by this dysregulation.

#### Menkes diseases

##### Genetic defects

The physiological importance of Cu-ATPases in humans can be illustrated by the deleterious consequences of the Cu-ATPase inactivation on cell metabolism. Mutations or deletions in the *ATP7A* gene encoding the Cu-ATPase ATP7A are associated with a fatal childhood disorder, Menkes disease. The Menkes gene is located on the long arm of the X chromosome at Xq13.3, and the gene product, ATP7A, is a 1500–amino acid P-type adenosine triphosphatase (ATPase) that has 17 domains—6 copper binding, 8 transmembrane, a phosphatase, a phosphorylation, and an ATP binding. The lack of functional ATP7A is associated with severe connective tissue defects in Menkes disease patients, likely due to disrupted delivery of Cu to lysyl oxidase in the secretory pathway. The connective tissue defects, including vascular tortuosity, loose skin, hyperextensible joints and bone fragility, are commonly observed in Menkes disease [48].

##### Pathophysiology of bone phenotype

Cu has an essential role in the normal maturation of collagen, particularly in the important steps to the formation of lysine-derived cross-link skeletal findings in infants with Menkes disease, the most characteristic of which are metaphyseal spurs, long-bone fractures, and wormian bones [52–54]. The long-term findings have been described by Amador et al. [52] and differ completely from those initially observed. In particular, undertubulation and metaphyseal flaring, similar to the findings seen in some types of bone dysplasia, are present [52]. In addition, the defective collagen cross-linking leads to osteoporosis and pathological fractures in these children [53, 54].

#### Therapy

Ceruloplasmin, the circulatory blue Cu protein, possesses antioxidant, cardioprotective and neuromodulatory properties. So far, subcutaneous Cu-histidine supplementation is the current choice of therapy, and long-term administration is not desirable because of the expected nephrotoxicity. Recently, a patient affected by Menkes disease was treated with weekly intravenous Cu-chloride and tolerated the procedure well with no major adverse events. However, optimum management of long-term survival of classical Menkes disease patients is currently unknown and a closed follow-up is mandatory for clarification of this phenotype [55]. Esmaeili et al. [56] described the preparation and characterization of four low molecular weight copper complexes, with special emphasis given to aspects related to the stability of these complexes in physiologically relevant media, to their potential antioxidant activity and to their biocompatibility with neuronal cells in view of a possible use in the treatment of neurological diseases. Furthermore, the study of these complexes may help to improve chelation therapy for Cu dysfunctions and to better understand Cu metabolism in humans [56].Changes in bone mineral density following pamidronate treatment have been described with 34–55% and 16–36% increases in lumbar spine bone mineral content and areal mineral density, respectively [54].

#### Wilson disease

##### Genetic defects

Mutations in the gene encoding the Cu-ATPase ATP7B also result in a severe metabolic disorder, known as Wilson disease (WD), which is inherited in an autosomal recessive manner. The *ATP7B* gene is approximately 80 Kb, located on human chromosome 13, and consists of 21 exons. The mRNA transcribed by the *ATP7B* gene encodes a protein of 1465 amino acids [57]. This protein is part of the P-type ATPase family, a group of proteins that transports metals into and out of cells by using energy stored in the molecule adenosine triphosphate (ATP). Cu-transporting ATPase 2 is found primarily in the liver, with smaller amounts in the kidneys and in the brain. WD is caused by various mutations in the *ATP7B* gene. In Western populations, the H1069Q mutation is present in 37–63% of cases, while in China this mutation is very uncommon and R778L is found more often. Ten percent of patients have no detectable mutations in the *ATP7B* gene [58].

##### Pathophysiology of the bone phenotype

Skeletal changes have been reported in WD, including osteoporosis [59, 60]. It is typically associated with an accumulation of Cu in various tissues, including bone tissue, with skeletal Cu content reported to be increased by about four times [60]. In a study by Hegedus et al., beta-cross-laps, a marker of bone remodeling, were found to be significantly increased in WD patients, suggesting increased bone resorption as a key mechanism contributing to bone loss and osteoporosis in these patients [61]. Weiss et al. [59] showed that the severity of liver disease is unrelated to the degree of bone loss as assessed by DXA, hypothesizing that Cu accumulation may directly affect bone metabolism. Indeed, accumulation of Cu causes production of reactive oxygen species (ROS) which is a risk factor for osteoporosis and may accelerate the effect of aging of bone by stimulating osteoclast differentiation and bone resorption and inhibiting osteoblast differentiation [59, 62]. In addition, an excess of Cu is able to induce mitochondrial dysfunction and mitochondrial toxicity, which are causally related to bone demineralization [59].

##### Therapy

In guidelines for the American Association for the Study of Liver Diseases, medical therapy is divided into 2 categories: treatment of symptomatic patients and treatment of asymptomatic patients [63]. Treatment of symptomatic patients (hepatic, neurologic, or psychiatric) should include chelation therapy, either with D-penicillamine or with trientine that increases urinary Cu excretion. Patients with WD are counseled to eat a diet low in Cu and avoid the very high Cu foods, such as mushrooms, chocolate, liver, and nuts [63]. Studies with sodium tetrathiomolybdate for the treatment of WD suggested some benefit for its use as initial treatment of patients with neurological complications [63]. A phase 2 study was conducted with a more stable form of a chelator for Cu, the choline tetrathiomolybdate (WTX101) molecule, that has the potential to increase biliary Cu excretion as well as bind Cu and albumin in an inert complex [64, 65]. Liver transplant can be used to rescue those with acute liver failure or those with liver disease who fail to respond to medical therapy. New advances in treatment may improve adherence and reduce some complications of treatment [64, 65].

Table 2 describes the liver diseases due to the alterations in the Cu metabolic pathway.

## Respiratory rare disease

### Description of the systemic disease

Rare lung diseases comprise many disorders and are described in more detail in the recent Orphan Lung Diseases Taxonomy by the European Respiratory Society [66]. In the present paper we describe cystic fibrosis, as the most relevant rare lung disease with an impact on bone metabolism.

#### Cystic fibrosis

Cystic fibrosis is an inherited disease characterized by the buildup of thick, sticky mucus that can damage many of the body's organs. The disorder's most common signs and symptoms include progressive damage to the respiratory system and chronic digestive problems. The features of the disorder and their severity varies among affected individuals [67].

##### Genetic defects

Mutations in the cystic fibrosis transmembrane conductance regulator (*CFTR*) gene cause cystic fibrosis [68]. The C*FTR* gene is localized on chromosome 7q31.2 and provides instructions for making a channel that transports negatively charged particles called chloride (Cl) ions into and out of the cells [69].

Cl is a component of sodium chloride, a common salt found in sweat, but it also has important functions in cellular metabolism, as the flow of Cl ions helps to control the movement of water in tissues [69]. Mutations in the CF transmembrane conductance regulator (*CFTR)* gene disrupt the function of the Cl channels, preventing them from regulating the flow of Cl ions and water across cell membranes. As a result, living cells in lungs, pancreas and other organs produce mucus that is unusually thick and sticky [69–71]*.*

##### Pathophysiology of the bone phenotype

Major pathogenic mechanisms mediating the development of CF bone disease (CFBD) in patients with CF may result from a combination of episodes of low bone turnover and formation rate during periods of disease stability, and an increased bone turnover and resorption during infective exacerbations [72]. Osteoporosis and increased vertebral fracture risk have been associated with CF disease, becoming more relevant with the increase in life expectancy of these patients. The first report on association of low BMD with CF was published in 1979 [71]. The etiology of low bone density is multifactorial, most probably a combination of inadequate peak bone mass during puberty and increased bone loss in adults [72]. Body mass index, male sex, advanced pulmonary disease, malnutrition and chronic therapies are established additional risk factors for CFBD [72]. Fractures not only cause pain and disability, but vertebral fractures and kyphosis further impair breathing biomechanics and often disqualify a patient from lung transplantation [73, 74]. Stalvey et al. developed and showed a CF disease model in which CFTR expression in bone directs reduced osteoblast differentiation and enhanced osteoclastic bone resorption [73]. This report, and other studies, demonstrate that CFTR is expressed in mesenchyme-derived osteoblasts, odontoblasts, chondrocytes and myocytes, and that mutations directly perturb cellular activities of these cell types [75, 76]. The in vitro evidence of delayed osteoblast new bone formation, and enhanced osteoclastic bone resorption resulting from reduced osteoprotegerin expression parallels the uncoupled bone turnover that is characteristic of CFBD in humans and in animal models [77, 78]. In addition, in human osteoblasts obtained from a 25-year-old CF male with the F508del/G542X mutation in the *CFTR* gene, a defective CFTR-mediated Cl-channel activity and a severe deficit in the production of osteoprotegerin  was described [77, 78]. These data raised the hypothesis that the *Cftr* gene may play a role in cell differentiation and bone matrix maturation [79].

##### Therapy

The goals of treatment primarily include: 1. preventing and controlling lung infections; 2. controlling of airway inflammation; 3. reducing viscoelasticity by removing thick, sticky mucus from the lungs and dilating the airways A new group of drugs called CFTR modulators are available which are able to correct the basic defect in CF, with an improvement in the management of the disease [75, 80]. However, most of these drugs are having serious hepatic and extra-hepatic side effects, in addition to high costs. Gene engineering techniques and new molecular targets may be explored besides CFTR. With the help of modern biology approaches like DNA nanotechnology, systems biology, metabolomics, disease modeling, and intracellular protein kinetics new pathways and networks associated with CF can be unraveled and eventually new therapeutic targets can become available [80, 81].

Table 3 describes the phenotypic characteristics of cystic fibrosis.

## Hematological rare disoders

### Description of the systemic disease

Rare blood disorders include various myeloproliferative diseases (also called myeloproliferative neoplasms), as well as histiocytosis and paroxysmal nocturnal hemoglobinuria (PNH). There are several kinds of myeloproliferative disease, including polycythemia vera, myelofibrosis, essential thrombocythemia, mastocytosis, and eosinophilia [82]. In a seminal paper Teti A. et al. described congenital bone diseases and their relationship to hematopoietic tissue [83]. Some of these diseases affect primarily the bone tissue and may have hematological alterations. These disorders have been also previously described by our group in the taxonomy of the rare metabolic skeletal diseases [3]. Here, we take in consideration the congenital hematological disorders that can be associated to a modification of bone turnover**.** They are represented by mastocytosis, beta-thalassemia, hemophilia type A and B, sickle cell disease, Ghosal hemato-diaphyseal dysplasia, severe congenital netropenia, and histiocytosis.

#### Mastocytosis

##### Description of the systemic disease

Mastocytosis is a complex disorder characterized by the accumulation of abnormal mast cells (MCs) in the skin, bone marrow and/or other visceral organs. The clinical manifestations result from MC-derived mediators and, less frequently, from destructive infiltration of MCs. Patients suffer from a variety of symptoms, including pruritus, flushing and life-threatening anaphylaxis. While mastocytosis is likely to be suspected in a patient with typical skin lesions (i.e. urticaria pigmentosa [UP]), the absence of cutaneous signs does not rule out the diagnosis of this disease. Mastocytosis should be suspected in cases of recurrent, unexplained or severe insect-induced anaphylaxis or symptoms of MC degranulation without true allergy [84].

In rare cases, unexplained osteoporosis or unexplained hematological abnormalities can be the underlying features of mastocytosis, particularly when these conditions are associated with elevated baseline serum tryptase levels. The diagnosis is based on the World Health Organization (WHO) criteria, in which serum tryptase levels, histopathological and immunophenotypic evaluation of MCs and molecular analysis are crucial [85]. The WHO classification subdivides mastocytosis into seven major categories: (1) cutaneous mastocytosis, (2) indolent systemic mastocytosis, (3) systemic mastocytosis with associated clonal, hematological non-mast-cell lineage disease, (4) aggressive systemic mastocytosis, (5) mast cell leukemia, (6) mast cell sarcoma, and (7) extracutaneous mastocytoma. Within these major categories additional variants and subvariants coexist, some of which are currently considered ‘provisional entities’ [85].

##### Genetic defects

Mastocytosis is associated with a somatic gain of function point mutation in the *KIT* gene. This was firstly recognized in 1995, when Nagata et al. [86] identified a point mutation in exon 17, resulting in a substitution of valine for aspartic acid in the catalytic domain of the *KIT* gene (D816V) in the peripheral blood of four patients with mastocytosis. Further analysis of larger cohorts confirmed that the *KIT* gene D816V mutationis detected in up to 93% of adult patients with the disease [84]. By contrast, patients with childhood onset mastocytosis may not have detectable *KIT* mutations or may express *KIT *mutations other than D816V (in exons 8, 9, 11 or 17) [84].

##### Pathophysiology of the bone phenotype

Osteopenia/osteoporosis with bone pain and possible pathological fractures has long been recognized in systemic mastocytosis (SM) patients. Half of adult patients with SM show bone involvement. Osteoporosis is the most prevalent bone manifestation in SM (31%) [87]. Serum Interleukin 6 (IL-6) is elevated in mastocytosis patients and correlates with severity of symptoms and the presence of osteoporosis. High serum IL-6 may not only signify disease progression, but may also participate in the pathophysiology of SM. The pathological fractures are considered a marker of aggressive SM, especially when associated with high levels of serum tryptase [84]. The main actor is the osteoclast with a relative or absolute predominance of bone resorption versus formation. Among the stimuli that drive osteoclast activity, the most important is the Receptor Activator of Nuclear Factor κ B (RANK)—RANK-Ligand (RANKL) signaling, but also histamine and other cytokines play a significant role in the process [84].

##### Therapy

During the last two decades, major discoveries contributed to a better diagnosis, identification of the clinical and biological abnormalities, and a refined classication of the different forms of mast cell disease [84]. Various cytoreductive treatments have been used for advanced SM, including 2-chlorodeoxyadenosine (2-CDA), interferon-alpha (IFN-α), classical chemotherapy agents (such as cytarabine or udarabine), but all with modest and disappointing response rates, highlighting the need for innovative therapies [84]. Tyrosine kinase inhibitors (TKIs) are an attractive therapeutic approach, given the pathogenesis of SM and the involvement of KITD816V mutation in more than 80% of patients, and other KIT mutations that map to the TK juxtamembrane domain or transmembrane domain in sporadic cases of SM. Many studies have reported quantitative and qualitative defects of signal transduction in SM [88]. These altered pathways play a role in the pathogenesis of SM and targeted drugs may provide therapeutic options by selective inhibition of some of these critical targets. Finally, normal and neoplastic mast cells express on their surface a number of cell surface antigens that might be considered as potential targeted therapies in advanced SM [88]*.* The central role of osteoclasts made bisphosphonates, as anti-resorptive drugs, the standard of treatment for bone involvement in SM [89]. Bisphosphonate therapy seems to improve lumbar spine BMD during SM-related osteoporosis. Spine x-ray and BMD should be performed in all SM patients to detect those who may benefit from an osteoporosis therapy [89].

#### Thalassemia

##### Description of the systemic disease

Thalassemia is an inherited blood disorder characterized by a defect in the globin chain synthesis in red blood cells. The clinical phenotype results from both the diminished amount of the particular globin chain as well as from the resultant chain imbalance that occurs because of normal production of the other globin chain [90]. The common forms include alpha (α) and beta (β) thalassemia [90]. In β thalassemia, the synthesis of normal α globin chains from the unaffected α globin genes continues as normal, resulting in the accumulation within the erythroid precursors of excess unmatched α globin [91]. In α thalassemia many mutations can affect the α globin gene, but the most common are gene deletions [91]. Anemia in β thalassemia results from a combination of ineffective erythropoiesis, peripheral hemolysis, and an overall reduction in hemoglobin synthesis. The severity of disease in β thalassemia correlates well with the degree of imbalance between α and non-α globin chains and the size of the free α chain pool. Thus, factors that reduce the degree of chain imbalance and the magnitude of α chain excess in the red cell precursors will have an impact on the final phenotype [91]. It might be important to note that the clinical phenotype becomes most apparent at 6–9 months of age, due to the fetal to adult hemoglobin switch that occurs at that age [90]. Finally, β thalassemia is the only form of this disorder where bone metabolism is involved.

##### Genetic defects

Thalassemia results when mutations affecting the genes involved in hemoglobin (Hb) biosynthesis lead to decreased Hb production. Depending on the mutation of genes located on chromosome 16 and 11 we can distinguish α and β thalassemia, respectively [90]. Mutations in the *HBB * gene cause β thalassemia. The *HBB * gene provides instructions for making beta-globin a subunit of hemoglobin. Some mutations in the *HBB* gene prevent the production of β globin [91]. About 100 mutations have been described that decrease β chain synthesis. Mutations fall into two classes: B0 refers to mutations that cause no β globin to be produced and B+ describes mutations that result in a diminished but not absent quantity of β globulin. The severity of these mutations can vary depending on the amount of normal β globin that is produced [91]. Depending the class of mutation present and the gene dosage (i.e. heterozygous or homozygous), patients can present with different severity of the disease [90]: (a) β thalassemia major (TM) refers to a severe clinical phenotype; (b) β thalassemia intermedia is characterized by a clinical phenotype based on heterogenous genetic mutations that still allow for some β chain production (e.g. B + /B0, B + /B +); and (c) β thalassemia minor/ thalassemia trait shows a mild clinical phenotype with a normal copy of the β globin gene being present (e.g. B + /B, B0/B) [90].

##### Pathophysiology of the bone phenotype

Metabolic bone disease represents a major cause of morbidity in patients with TM (the most severe form). The pathogenic factors include: the primary disease itself that causes bone marrow expansion, the chronic anemia, and the iron endocrine toxicity [92, 93].

Marrow expansion causes mechanical interruption of bone formation, leading to cortical thinning and increased distortion and fragility of the bone [93]. Other factors lead to alterations in the RANK/RANKL/osteoprotegerin system, increasing osteoclastic activity and enhancing osteoblast dysfunction [94, 95]. The increase of RANKL, followed by unmodified osteoprotegerin levels, with the consequent increase of RANKL/osteoprotegerin ratio, may represent the cause of the uncoupling of bone turnover observed in thalassemia patients. However, no correlation between sRANKL or osteoprotegerin levels with BMD of the lumbar spine or at the femoral neck is present [93].

Hypogonadotrophic hypogonadism and delayed puberty are the most common endocrine complications in patients with TM, and they also contribute to osteopenia and osteoporosis. Morabito et al. [96] showed a negative correlation between RANKL and free testosterone in male thalassemia patients and with 17β-estradiol in female thalassemia patients, which suggests that a reduced production of sex steroids causes an increase in RANKL production [96]. There are gender differences not only in the prevalence but also in the severity of the osteoporosis syndrome. The reported frequency of osteoporosis, even in well-treated TM patients, varies from 13.6% to 50% with an additional 45% affected by osteopenia [92]. The lack of an anabolic effects of growth hormone (GH) and insulin-growth factor I (IGF-1) on bone formation for the acquisition of bone mass, mainly during childhood and puberty, is clearly involved in the pathogenesis is of bone mass reduction in thalassemic patients.

##### Therapy

Transfusion therapy works by supplying normal erythrocytes and suppressing ineffective erythropoiesis, essentially controlling all downstream pathophysiological mechanisms in thalassemia. Whether iron overload develops from increased intestinal absorption or secondary to regular transfusions, it can cause substantial morbidity and mortality, and so warrants prompt diagnosis and effective management. For this reason it is important to use iron chelators in the affected patients. Three iron chelators are currently available for the treatment of iron overload in patients with thalassemia: deferoxamine, deferiprone and deferasirox. Finally, splenectomies have traditionally been performed as an alternate or adjunct therapy to transfusion [93]. In TM, GH secretory dysfunction is common and contributes to osteopenia and osteoporosis, along with other endocrinopathies, such as hypoparathyroidism, vitamin D deficiency, hypothyroidism, and diabetes [94]. Prevention is without a doubt the first step in the management of osteoporosis in TM, with the final goal of preventing bone fragility and fractures. The management of patients with TM should start as early as at birth in order to minimize disease complications [85–94].

**Fig. 3:**
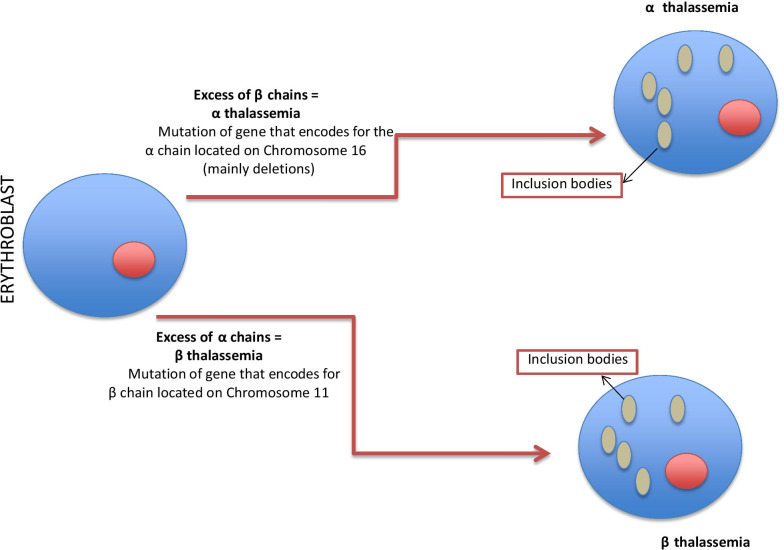
Pathogenesis of thalassemia. α Thalassemia: mutations of the gene encoding for the α chain located on chromosome 16 (mainly deletions) induces an excess of β chains with the presence of inclusion bodies in the cells. β Thalassemia: mutations of the gene located on chromosome 11 that encodes for the β chains induces an excess of α chains with the presence of inclusion bodies in the cells

Figure 3 shows the mechanisms responsible for the pathogenesis of thalassemia.

#### Hemophilia

##### Description of the systemic disease

Hemophilia is a male bleeding disorder with worldwide distribution. Affected individuals have either reduced or absent levels of coagulation factor VIII and IX in plasma (hemophilia A and hemophilia B respectively). Hence, they encounter unusual bleeding, either spontaneous or after trauma and surgery. The spectrum of bleeding manifestations varies from superficial ecchymosis to lethal hemorrhage in the central nervous system [97, 98]. While the prevalence of hemophilia A varies among different countries, its prevalence has been estimated to be about 3 to 20 per 100,000 in the population. Hemophilia B is seen as 1 case among 5,000 male births in European countries and the United States [97, 98]. Individuals with hemophilia A and B have respectively either reduced or absent levels of coagulation factor VIII and IX. Lack or reduced levels of coagulation factors VIII and IX result in impaired clot formation and tendency to bleeding episodes. These lifelong disorders are categorized as severe type (< 1%), medium type (1–5%), and mild type (5–30%)  [98]. Replacement therapy using plasma derived or recombinant coagulation factor concentrates are the basis of treatment for bleeding episodes and prophylaxis regimens [99].

##### Genetic defects

A X-linked, recessive hemorrhagic trait or gene induces Hemophilia A. Hemophilia A X-linked trait manifests as a congenital absence or decrease in plasma clotting Factor VIII, a pro-coagulation cofactor and robust initiator of thrombin that is essential for the generation of adequate amounts of fibrin to form a platelet/fibrin plug at sites of endothelial disruption. Female hemophilia gene carriers do not manifest symptoms of Hemophilia A but may have lower than usual quantities of Factor VIII. Male Hemophilia A patients do not transmit hemophilia to male offspring, but all their female offspring will carry the hemophilia gene.

##### Pathophysiology of the bone phenotype

Twenty-seven percent of hemophilic patients have osteoporosis and 43% osteopenia [92]. A large and growing body of literature has investigated osteopenia and osteoporosis both in children and in adults with hemophilia [98, 100, 101]. While physical activity is considered as a central indicator of bone resorption, patients with bleeding disorders are less likely to engage in high impact activities and weight-bearing exercises [98]. . Hemarthrosis, infection with hepatitis C virus are also commonly reported causes of susceptibility of hemophilia to reduced bone density [98]. Recently, a survey in hemophilia A, hemophilia B and von Willebrand disease (vWD) showed a higher risk of bone health outcomes in comparison with healthly controls and suggested a possible role of endogenous coagulation factors in the maintainanceof bone mass [102]**.**

##### Therapy

The first successful treatment of hemophilia A with whole blood transfusion was reported in 1840 and subsequently, treatment with plasma was introduced,  with the cryoprecipitate fractions of plasma enriched in FVIII firstly utilized in 1964 [97]. High purity Factor VIII concentrate treatment started in 1984 with the production of recombinant FVIII (rFVIII) generated through cloning of FVIII [103]. The development of recombinant FVIII (rFVIII) infusion has improved the life expectancy of patients with mild to moderate hemophilia A, reaching levels comparable to that of the general population. However, an ongoing concern is the development of inhibitory antibodies to plasma-derived FVIII or rFVIII. Prophylaxis appears to be used less frequently in hemophilia B than in hemophilia A patients.

Gene therapy clinical trials for hemophilia B, which is caused by the lack of another clotting protein Factor IX are ongoing. A gene therapy is more challenging for hemophilia B in comparison with hemophilia A, because of the molecular properties of FVIII. One of the major obstacles in the development of successful clinical trials of FVIII gene therapy has been the large size of the *F8* gene.

Transplantation and engraftment of liver sinusoidal endothelial cells (LSECs) capable of producing FVIII provides the most natural pathway for the cure of hemophilia A. Such a cell based therapy for hemophilia A offers the potential of a life-long cure if a number of hurdles can be overcome. Hemophilia B may have been relatively neglected compared to haemophilia A, but of note, major recent advances in haemophilia therapy (i.e. longer-acting factor concentrates with extended half-lives, and gene therapy) will potentially be available for haemophilia B before haemophilia A [104, 105]. For bone health, the results of a study on the effect of prophylaxis in hemophilia indicated that replacement therapy as a prophylaxis regimen beginning in early childhood may preserve normal BMD in severe hemophilia patients [106].

#### Sickle cell disease

##### Description of the systemic disease

Sickle cell disease (SCD) is an inherited disorder of hemoglobin (Hb) synthesis affecting many individuals throughout the world. SCD manifests itself soon after the protective effect of HbF diminishes. Its complications vary: acute chest syndrome, proliferative retinopathy, pulmonary hypertension, renal insufficiency, cerebral vascular accident, and musculoskeletal complications [107]. SCD is classified by the number of globin genes that are affected and inherited in an autosomal recessive manner. The heterozygous form is when the individual only carries one gene for HbS and is called sickle cell trait. It occurs frequently in individuals of African ancestry with 8% to 10% of African Americans carrying the trait. This is benign and the affected individual will not show any symptoms and no treatment or precautions are needed. The homozygous state is the most common and severe form of SCD and is called sickle cell anemia (HbSS), accounting for 60–70% of SCD in the United States (US). In HbSS, the red blood cells are distorted and sickle shaped [107]. The two major characteristics are chronic hemolytic anemia and intermittent vaso-occlusion that results in tissue ischemia and causes acute, severe pain episodes. SCD is a chronic disease that has detrimental effects on the entire body [107].

##### Genetic defects

SCD encompasses a group of disorders characterized by the presence of at least one hemoglobin S allele (*HbS*; p.Glu6Val in HBB) and a second HBB pathogenic variant resulting in abnormal hemoglobin polymerization. HbS/S (homozygous p.Glu6Val in HBB) accounts for 60–70% of SCD in the United States. Other forms of SCD result from coinheritance of HbS with other abnormal βglobin chain variants. SCD is inherited in an autosomal recessive manner. If one parent is a carrier of the HBB *HbS* pathogenic gene variant and the other is a carrier of any of the HBB pathogenic variants (e.g., HbS, HbC, β thalassemia), each child has a 25% chance of being affected, a 50% chance of being an unaffected carrier, and a 25% chance of being unaffected and not a carrier. Prenatal diagnosis for pregnancies at increased risk for SCD is possible by molecular genetic testing if the HBB pathogenic variants have been identified in the parents [107].

##### Pathophysiology of bone phenotype

The bone involvement in SCD ranges from acute manifestations, such as painful vaso-occlusive crisis or osteomyelitis, to more chronic and debilitating complications, such as osteonecrosis, osteoporosis and osteopenia, impaired growth and chronic infections [107]. Although these bone complications may not contribute directly to mortality, they are the major source of morbidity with a high impact on patients’ quality of life [107]. Osteopenia and osteoporosis are often asymptomatic but may cause pain, fractures, deformity, and vertebral collapse [107–109].

Serrai et al. [110] observed a high prevalence (76%) of abnormal BMD (low and very low) by DXA in SCD adults, with predilection for the lumbar spine, and they found a strong correlation between low Hb level and abnormal BMD [110]. Chronic and severe anemia places a burden on the bone marrow, with increased erythropoiesis causing hyperplasia of the bone marrow, decrease in the trabecular network, osteopenia and bone destruction [110]. In addition, various micronutrient and macronutrient deficiencies leading to delayed skeletal maturation (malnutrition, vitamin D and zinc deficiency, etc.), hormonal deficiency (reduced GH, hypogonadism), and poor weight bearing because of chronic pain and inactivity, may all contribute to the development of sickle bone disease [108, 109]. Over 70% of adults with SCD will develop low BMD, which typically occurs 2–3 decades earlier (median age 30 years) than in the general population [108].

##### Therapy

Hematopoietic stem cell transplantation has the potential to establish normal erythropoiesis with stable long-term engraftment, amelioration of symptoms, and stabilization of organ damage. Early results in gene therapy for SCD are encouraging. Evaluation of the long-term benefits of curative therapies for SCD requires comparative clinical trials and studies of late effects of transplantation [111].

#### Ghosal type hemato-diaphyseal dysplasia

##### Description of the systemic disease

Ghosal hematodiaphyseal dysplasia is a rare inherited condition characterized by abnormally thick bones and a shortage of red blood cells [112]. It is an autosomal recessive disorder characterized by metadiaphyseal dysplasia of the long bones and defective hematopoiesis due to fibrosis or sclerosis of the bone marrow [112]. The disease was described for the first time by SP Ghosal in five children who had moderate to marked anemia and diaphyseal dysplasia [112]. Hematologic manifestations of the syndrome include varying degrees of anemia, thrombocytopenia, or pancytopenia [112]. Only a few cases have been reported in the medical literature. Most affected individuals have been from the Middle East and India. Treatment consists of steroid therapy and most cases reported in literature previously required a low-dose maintenance therapy throughout life to keep hemoglobin at normal levels [112].

##### Genetic defects

Ghosal hematodiaphyseal dysplasia results from mutations in the thromboxane synthase *TBXAS1* gene localized on chromosome 7q34. This gene encodes thromboxane-A-synthase, an enzyme which produces thromboxane A2(TA2). TA2 is responsible for platelet aggregation (via the arachidonic acid cascade) and also modulates bone mineral density variation (by influencing the expression of *TNFSF11* and *TNFRSF11B*, which encode RANKL and osteoprotegerin in osteoblasts, leading to bone sclerosis). Most reported mutations in the *TBXAS1* gene are of missense type and the pathogenesis of the bony and hematological abnormalities are presumed to be due to a sclerosing bone dysplasia secondarily leading to marrow hypocellularity [112].

##### Pathophysiology of the bone disease and the bone phenotype

In a Caucasian patient with two biallelic mutations within the gene *TBXAS1* (c.266 T > C; c.989 T > C) bone densitometry revealed increased bone density with Z-score of 4.5, consistent with mild thickening of long bone diaphysis noted via X-ray [113, 114]. Strikingly, the bone marrow biopsy revealed haphazard ossification, immature cartilage formation and thickened trabeculae replacing hematopoiesis within the marrow cavity [112, 113]. *TBXAS1* defects decrease TXA2-mediated osteoblast RANKL expression paralleled by elevated production of osteoprotegerin, ultimately inhibiting osteoclast differentiation [113]. In addition, increased PGE2 synthesis may induce inflammation and increase bone density [113]. Similar to the Ghosal type hemato-diaphyseal dysplasia is the Camurati-Engelmann disease, an extremely rare disease characterized by hyperostosis of multiple long bones caused by mutations in the *TGFB1* gene. However, in the Camurati-Engelmann disease, only the diaphysis is affected, while both diaphysis and metaphysis are affected in Ghosal type hemato-diaphyseal dysplasia [83].

##### Therapy

No specific therapy exists.

#### Severe congenital neutropenia

Severe congenital neutropenia (SCN) patients are affected by congenital severe neutropenia, with an absolute neutrophil count (ANC) of less than 0.2 × 10^9^/L [115]. Bone marrow examination in the majority of cases reveals a maturation arrest of myelopoiesis at the level of promyelocytes, which generally leads to reduced neutrophil counts but increased numbers of atypical promyelocytes. In these patients, the risk of infections such as otitis, gingivitis, skin infections, pneumonia, deep abscesses and septicemia begins in the neonatal period [115]. Furthermore, patients with severe congenital neutropenia have an increased risk of developing leukemia [115]*.* The prevalence of SCN is estimated to be 3–8.5 cases per million individuals. Prevalence depends on several factors: the success of the mother’s pregnancy, the frequency of neonatal deaths from infections or other causes, the pattern of inheritance for the specific genetic disorder, and the ascertainment of the diagnosis through measurement of the blood ANC early in life [116].

##### Genetic defects

Demographics play an important part in epidemiology. Worldwide, autosomal dominant disorders seem to be more common, whereas the recessive disorders are usually diagnosed in consanguineous kindreds [115]. Several genes can be involved in severe congenital neutropenia and play a role in the maturation and function of neutrophils, promoting cell survival and response to immune signals. Most patients (about 50%) harbor mutations of the *ELANE* gene [83], encoding the neutrophil elastase, while about 10% of patients instead harbor mutations in the *HAX1* gene, encoding the HS-1-associated protein X-1 (HAX1), where HS-1 is a Src family tyrosine kinase substrate and HAX1 is a mitochondrial protein that regulates apoptosis [83]. HAX1 protein contributes to the activation of the granulocyte colony-stimulating factor (G-CSF) signaling pathway and could cause osteopenia enhancing bone resorption [83]. Various other genes, including *CSF3R, G6PC3, GFI1, JAGN1, TCIRG1, VPS45* and *WAS*, account for a small portion of patients, while about 30% have unknown genetic defects, with some cases with no familial history, thus classified as sporadic [117]. The *GFI1* gene encodes a transcription factor that controls human stem cells quiescence and osteoblast differentiation, inducing epigenetic changes in the RUNX2 promoter [83].

##### Pathophysiology of the bone phenotype

SCN is associated with osteopenia in at least 40% of patients, which can then evolve towards overt osteoporosis. Recurrent infections and diminished physical activity may play a prominent role in decreasing BMD because of increased catabolism and poor nutritional status [116]. On the other hand, exogenous administration and genetically induced overexpression of G-CSF have been related to bone mineral loss in animal studies [116]. On the basis of the evidence that osteoclasts are derived from the monocyte/macrophage lineage, various hypotheses state that a large group of cytokines and CSFs involved in hematopoiesis, including G-CSF, regulate osteoclast development and activity [108]. The receptor activator of nuclear factor-kB (RANK), the RANK-L, and osteoprotegerin. are important regulators of osteoclastogenesis [116]. Elhasid et al. [118] described a 4-year-old patient who had familial SCN and severe osteoporosis with multiple fractures that presented very low levels of osteoprotegerin with a high RANKL/osteoprotegerin ratio. This may play an important role in the pathogenesis of osteoporosis in these patients [118].

##### Therapy

No specific therapy is available. The anemia can be treated with oral prednisolone with walking difficulties improvement [86]. Bisphosphonates have been used for the treatment of bone loss [116].

#### Histiocytosis

##### Description of the systemic disease

Histiocytoses are a diverse group of hematologic disorders defined by the pathologic infiltration of normal tissues by cells of the mononuclear phagocyte system (MPS). Cells of the MPS have a wide range of morphologic, anatomic and functional characteristics that make classification of this system difficult. The development and differentiation of the cells of the MPS, much like other hematopoietic lineage, is driven by a tightly regulated pattern of gene expression governed by distinctive sets of transcription factors that control cell proliferation and differentiation. As proposed in 1987 [119], histiocytic disorders can be classified into three classes based on the pathologic cells present within the lesions:

- Class I: Langerhans cell histiocytoses and other dendritic cell disorders;
- Class II: non-Langerhans cell histiocytoses primarily consisting of hemophagocytic lymphohistiocytosis;
- Class III: malignant histiocytosis

This system was revised in 1997 by the WHO Committee on Histiocytic/Reticulum Cell Proliferations and the Reclassification Working Group of the Histiocyte Society [120]. The central theme of this reclassification effort consisted of distinguishing the clearly malignant histiocytoses from the remaining subtypes, the so-called “disorders of varied biological behavior” [121].

#### Langerhans cell histiocytosis (X-Histiocytosis)

Langerhans cell histiocytosis (LCH) is a group of idiopathic disorders characterized by the presence of cells with characteristics similar to bone marrow–derived Langerhans cells juxtaposed against a backdrop of hematopoietic cells, including T-cells, macrophages, and eosinophils. It is a rare disease of unknown pathogenesis, leading to its high rate of misdiagnosis and missed diagnosis.

LCH is divided into 3 entities: Letter-Siwe disease, Hand-Schuller-Christian disease, and eosinophilic granuloma [121]. LCH can occur at any age, but mainly in children of 1 through 4 years of age. The incidence of LCH in adults is 1–2 cases per million. Most LCH patients are males. The sex ratio (m:f) is 2:1 [121]

##### Genetic defects

Somatic mutations in the *BRAF* gene have been identified in the Langerhans cells of about half of individuals with Langerhans cell histiocytosis [119]. Somatic gene mutations are acquired during a person's lifetime and are present only in certain cells. These changes are not inherited. A major breakthrough in the LCH puzzle came with discovery of recurrent somatic *BRAF* V600E gene mutations in histiocytes in 50% of LCH lesions, 13 of which have been validated in multiple subsequent studies [122]. BRAF is a central kinase of the MAPK pathway (RAS/RAF/MEK/ERK) that transduces extra-cellular signals and regulates critical cellular functions [122]. The V600E mutation results in *RAS*-independent constitutive activation of downstream *MEK* and *ERK* [118]. Besides the V600E mutation, single case reports have described additional somatic mutations within the *BRAF* gene locus (*BRAF* V600D, *BRAF* 600DLAT), as well as the germline mutation/polymorphism *BRAF* T599A with potential functional consequences [122].

##### Pathophysiology of the bone phenotype

The skeletal system is the most common site of involvement of Langerhans cell histiocytosis, and in 60–80% of cases is the only organ system involved. The lesions may be asymptomatic and discovered as an incidental radiographic finding, when symptomatic patients complain pain, swelling and tenderness around the lesion. Systemic symptoms may also be present, including general malaise and, on occasion, fever with leukocytosis [121, 123]. Makras et al. [124] in a retrospective study examined whether BMD and indices of bone turnover are affected in adult patients with LCH and whether these patients are at high risk of developing osteoporotic fractures. Their findings are particularly relevant as such patients are probably at high risk of developing osteoporosis due to the secretion of bone resorbing cytokines from the pathological process and the presence of a number of several predisposing factors such as anterior pituitary deficiencies and treatment with glucocorticoids and/or chemotherapy. Indeed, adults with LCH have high serum osteoprotegerin levels and low serum RANKL levels than controls [125]. In addition, RANKL is abundantly expressed in cells of adult LCH lesions from different tissues, especially within inflammatory infiltrates [125] and concomitant nuclear staining of p65 NFκB, is associated with increased RANKL expression, suggesting that RANKL could be directly involved in any lesional cell activation [125].

##### Therapy

LCH has a wide spectrum of clinical presentations and variable outcomes. With current risk-adapted treatments, more than 80% of patients are cured. However, 30% to 50% of patients experience disease recurrence and a significant number of survivors develop neurodegeneration. Treatments aimed at achieving long-term cures with low reactivation rates and that could prevent the development of neurodegeneration need to be developed. Targeted therapies with BRAF or MEK inhibitors offer the possibility of reframing the treatment of LCH in the near future [126]. Denosumab administration, a RANKL inhibitor, seems a rational treatment strategy in LCH in order to enhance further endogenous osteoprotegerin action and interrupt the lesional immunological process if RANKL related. Makras P et al. showed that the administration of denosumab in patients affected by adult LCH resulted in a significant remission of the disease activity at both osseous and nonosseous lesions [127]. The Authors concluded that denosumab could be considered an effective treatment option in adults with multisystem LCH, also exerting a significant analgesic effect on bone lesions [127, 128].

#### Non-Langerhans cell histiocytosis: Erdheim-Chester disease (ECD)

Erdheim-Chester disease (ECD) is a rare, non-Langerhans histiocytosis described by Jakob Erdheim and William Chester in 1930 [129]. Historically, ECD has been considered a variably aggressive histiocytic disorder of unclear origin with poor response to therapy. However, the identification of the clonal nature of the disorder with at least one therapeutically relevant recurrent oncogenic somatic mutation has reformulated our understanding of the pathogenesis and clinical management of the disease [129].

Identifying distinctive histopathologic findings in the appropriate clinical and radiologic context makes possible to evidence infiltration of typically foamy or lipid-laden histiocytes with admixed or surrounding fibrosis [130]. Touton giant cells are often present. At the immunohistochemical staining, ECD histiocytes are positive for CD68, CD163, and Factor XIIIa, and negative for CD1a and langerin (CD207), with positivity for S100 being observed rarely[130]. This differentiates ECD from LCH, where Langerhans cells are positive for CD1a, S100, and langerin [130]. In addition to histological features, the radiographic finding of symmetric diaphyseal and metaphyseal osteosclerosis in the legs is nearly always present in ECD [130].

It is a disease with almost constant bone involvement and an extraosseous involvement only in 60% of patients [131]. The pattern of extraosseus clinical manifestations is highly variable and may be life-threatening [131].

#### Genetic defects

Recent discovery of  *BRAF* (V600E) gene mutations in LCH by Badalian-Very et al. [132] yielded the first identification of a bona fide oncogenic alteration in this disorder. Estimates of *BRAF*(V600E) gene mutation frequencies in ECD range between 38 and 68% in most reports, with one recent report suggesting that nearly 100% (18/18) of ECD patients have the mutation if sufficiently sensitive techniques are used [132]. In addition, an oncogenic *NRAS * (Q61R) gene mutation was identified in an ECD patient, further highlighting the importance of mitogen-activated protein kinase signaling to ECD pathogenesis [132]. To further support the clonal nature of ECD, ECD histiocytes have been found to express a pattern of proinflammatory cytokines and chemokines responsible for local activation and recruitment of histiocytes [132]. Based on these studies, ECD can now be defined as a clonal disordercharacterized by frequent hyperactivation of mitogen-activated protein kinase signaling in which an inflammatory milieu is important in the pathogenesis and clinical manifestations of the disease [132].

##### Pathophysiology of the bone phenotype

The most frequently affected bones are the femur, the tibia and the fibula and, less frequently, the ulna, the radius and the humerus. Bone pain usually manifests around the knees and ankles. Osteosclerosis occurs bilaterally and symmetrically in the diametaphyseal regions of the long bones. While the classical hallmark of the skeletal involvement of ECD is osteosclerosis, mixed sclerotic and lytic lesions have occasionally been described. Conversely, bone lesions found in LCH are lytic rather than sclerotic [133] and this complicates the diagnostic  iter. The distribution of sclerotic lesions in ECD differs from that of LCH in that the latter commonly involves the calvarium, facial bones, proximal limbs, pelvis, and scapula, rather than the distal limbs as in ECD [133].

##### Therapy

For decades, corticosteroids, cytotoxic agents such as vinca alkaloids and immunosuppressive agents (i.e. cyclophosphamide, methotrexate and azathioprine) represented the therapeutic milestone for ECD patients. More recently, interferon (IFN)-based therapy has emerged as a reliable option for ECD patients. Inhibition of BRAF activation by vemurafenib is a highly promising treatment, in particular for its ability to overcome the blood brain barrier. Mutations of *BRAF* gene result in a conformational change of a serine/threonine-protein kinase that leads to a chronic activation of the RAS-RAF-MEK-ERK pathway, therefore accelerating the proliferation of the cells [133]. Intriguingly, the recent discovery of additional *NRAS* gene mutations in ECD strongly suggests that the entire RAS-RAF-MEK-ERK pathway plays a pathogenic role in histiocytosis, thus raising interest in dual BRAF/MEK inhibition [134]. Improved understanding of the pathogenetic mechanisms underlying ECD will lead to the development of more effective targeted therapies, while an accurate stratification of patients based on their neurologic involvement will help clinicians to maximize the benefits of novel molecular targeting for therapies.

Table 4 describes the hematological disorders with bone involvement.

## Neurological rare diseases

Many neurological and neuromuscular diseases may cause an increase in the risk of osteoporosis and fragility fractures [135]. Although the etiology of osteoporosis is multifactorial (with genetic factors accounting for 70% of the variability in bone density) exposure to high doses of corticosteroids and poor mobility, conditions typical of some neurological or neuromuscolar diseases, are two important potential causes of bone fragility. In addition, epileptics are a separate group of patients at particular risk of fracture [135]. Here we selected Rett syndrome to describe the costellation of the bone phenotype, that characterizes this disorder neurological in nature.

### Rett syndrome

#### Description of the systemic disease

Rett Syndrome (RS) is a severe neurological disease, characterized by the arrest of brain development. Most individuals with RS express a mutation in the *MECP2* gene, which either activates or represses neural transcription when it binds to methylated cytosines in DNA [135, 136]. Clinical outcomes for this syndrome are complex, with varying degrees of autonomic dysfunction [137]. Patients with classic RS appear to develop normally until 6–18 months of age, then gradually lose speech and purposeful hand use, and develop microcephaly, seizures, autism, ataxia, intermittent hyperventilation, and stereotypic hand movements. After this initial regression, the condition stabilizes and patients usually survive into adulthood [137].

#### Genetic defects

As RS occurs almost exclusively in females, it has been proposed that it is caused by an X-linked dominant mutation with lethality in hemizygous males [137, 138]. Previous exclusion mapping studies using RS families mapped the locus to Xq28 [139]. In a systematic gene screening approach, mutations in the *MECP2* gene encoding the X-linked methyl-CpG-binding protein 2 (MeCP2) were recognized as the cause of some cases of RS [140]. MeCP2 selectively binds CpG dinucleotides in the mammalian genome and mediates transcriptional repression through interaction with histone deacetylase and the corepressor SIN3A [140].

#### Pathophysiology of the bone phenotype

RS patients also develop skeletal abnormalities such as scoliosis, low bone density and a high frequency of fractures [141–146], with a seriously impairement in the mobility and in the quality of life at a young age. Immobility, development of epilepsy and anticonvulsant medications are all factors that can contribute to the development of osteoporosis [95, 141].

#### Therapy

Pre-clinical research strongly suggests that RS could be one of the first curable neurological disorders. The application of genome-wide approaches and increasingly sophisticated model systems are painting a clearer picture in the functional role of MeCP2 in neurons, and this new information promises to guide future therapeutic strategies [141, 142]. Bisphosphonates constitute a beneficial adjuvant treatment to diminish the risk of fracture and restore bone density. In addition, anabolic treatment with teriparatide produces a great improvement in the bone microarchitecture in patients with RS [143, 144].

Table 5 describes the neurological disorders with bone involvement.

## Malformation Syndrome

### Thricho-rhino-phalangeal syndrome (Langer-Giedion Syndrome)

#### Description of the systemic disease

Trichorhinophalangeal syndrome (TRPS) is a rare, autosomal dominant malformation syndrome characterized by hair, craniofacial and skeletal abnormalities, skin laxity, deformation of phalanges and anomalies of pelvis, femurs, and tibias [144].

#### Genetic defect

The *TRPS1* gene (OMIM: 604386) is located on 8q24.12 and encodes for a zinc-finger (ZF) transcriptional repressor, Trps1. Trps1 binds DNA through a single GATA-type ZF that recognizes a DNA consensus sequence common to all GATA transcription factors. However, the repression activity of Trps1 maps to the C- terminal Ikaros-like double ZF motif [145]. Genotype – phenotype correlation analyses revealed that three distinctive clinical types of TRPS are associated with different kinds of the *TRPS1* gene mutations [145]. The mildest form, TRPS type I, is caused mostly by entire gene deletions and nonsense mutations located before the region coding for the DNA-binding domain. Missense mutations in the DNA- binding domain cause the more severe TRPS type III. TRPS type II (Langer-Gideon syndrome) combines features of TRPS type I and multiple exostoses and it is caused by contiguous deletion of the *TRPS1* and *EXT1* genes [145].

#### Pathophysiology of the bone phenotype

Previous studies identified the Trps1 transcription factor as a potentially novel transcriptional repressor both of the Runx2 promote, the master regulator of osteoblast differentiation and of chondrocyte hypertrophy [146]. Interestingly, during the development of endochondral bones, Trps1 is highly expressed in regions where Runx2 is downregulated [146]. The loss of repression of Runx2-ihh loop in *Trps1* knockout mice results in altered endochondral bone formation, which is characterized by dysregulation of chondrocyte differentiation and uncoupling of processes of perichondrial mineralization and chondrocyte maturation [146, 147]. Biphosponate infusional therapy seemed to be effective, both in terms of clinical (absence of new fractures) and densitometric parameters, and can be considered as a possible therapeutic option in case of bone fragility in patients with TRPS [147] (Table 6).

Table 6 describes the malformative syndrome.

**Table 6:** Malformative syndrome

Disease	OMIM phenotype number	OMIM gene/locus number	Gene	Chromosome location	Bone phenotype (specific signs and symptoms)	Bone biochemical markers
*Tricho-rhino-phalangeal syndrome, type I*	190350	604386	*TRPS1*	8q23.3	Craniofacial abnormalities and disturbances in formation and maturation of bone matrix. Clinodactyly, phalangeal epiphyses of the hands appearing as cone-shaped, short stature, hip joint malformations, joint pain (late-onset), osteopenia (late-onset), osteoarthritis (late-onset), scoliosis, lordosis, coxa plana, coxa magna, flattened capital femoral epiphyses, swelling of proximal interphalangeal joints, short metacarpals, short metatarsals, and pes planus, osteoporosis	NR

Bone biochemical markers acronyms present in the Tables

*BSAP* bone specific alkaline phosphatase, *NTX* corss-linked N-telopeptide of type I collagen, *OCN* osteocalcin, *DPD* deoxypyridinoline, *PYR* pyridinoline, *CTX* corss-linked C-telopeptide of type I collagen, *OPG* osteoprotegerin, *PTH* parathyroid hormone, *25 (OH) D*_*3*_ 25 hydroxyvitamin D_3,_ *IL-1α* interleukin 1 alpha, *TNF-α* tumor necrosis factor alpha, *IL-6* Interleukin 6, *P1NP* Procollagen type 1N-terminal propeptide

## Conclusions

The knowledge of the impact of non-skeletal diseases on bone metabolism is of paramount importance for the therapy of bone complications and can guide the clinician in the choice of the most appropriate pharmacological intervention. The changes in bone metabolism require a particular attention considering that, due to better targeted therapies, patients survive into adulthood and in many cases the loss of bone mass may be an important problem to decrease the quality of life. An adequate patient education in term of intake of calcium, vitamin D and physical activity should be recommended. Currently, the best advice is to treat osteopenia and/or osteoporosis according to general guidelines using anti-resorbitives or anabolic drugs. The evolution of drug development for osteoporosis, from clinical observations to the more recent framework of fundamental bone biology driving novel therapeutics, is truly remarkable. Several drugs can be used to improve bone mass and  to reduce fracture risk in osteoporosis. These drugs may have an antiresorbitive, an anabolic or a bone building effect on bone. The characterization of bone metabolism for the bone-forming or bone-resorbing phenotype in patients with non skeletal diseases in origin, will lead to different therapeutic approaches (e.g., anabolic, antiresorptive or bone building drugs).

## Data Availability

Not applicable.

## Abbreviations

IOF: International Osteoporosis Foundation
SRD-WG: Skeletal Rare Diseases Working Group
LSDs: Lysosomal storage diseases
GD: Gaucher disease ERT: enzyme replacement therapy
CBS: Cystathionine-β-synthase
HCY: Homocysteine
PLP: Pyridoxal 5′-phosphate
VDR: Vitamin D Receptor
BMD: Bone mineral density
ECM: Extracellular matrix
OA: Osteoarthritis
Phe: Phenylalanine
Cu: Copper
ATPase: Adenosine triphosphatase
WD: Wilson disease
CFTR: Cystic fibrosis transmembrane conductance regulator
CFBD: Cystic fibrosis bone disease
MCs: Mast cells
SM: Systemic mastocytosis
RANK: Receptor Activator of Nuclear Factor κ B
RANK-L: RANK-Ligand
TKIs: Tyrosine kinase inhibitors
Hb: Hemoglobin
TM: Thalassemia major
SCD: Sickle cell disease
HSCT: Hematopoietic stem cell transplantation
SCN: Severe congenital neutropenia
ANC: Absolute neutrophil count
ECD: Erdheim-Chester disease
IHC: Immunohistochemical
IFN: Interferon
RS: Rett syndrome
TRPS: Trichorhinophalangeal syndrome
BSAP: Bone specific alkaline phosphatase
NTX: Corss-linked N-telopeptide of type I collagen
OCN: Osteocalcin
DPD: Deoxypyridinoline
PYR: Pyridinoline
CTX: Corss-linked C-telopeptide of type I collagen
OPG: Osteoprotegerin
PTH: Parathyroid Hormone
[1,25(OH)_2_D_3_]: 1,25-Dihydroxy vitamin D_3_
25 (OH) D3: 25 Hydroxyvitamin D3
IL-1α: Interleukin 1 alpha
TNF-α: Tumor necrosis factor alpha
IL-6: Interleukin 6
P1NP: Procollagen type 1N-terminal propeptide
